# A Metaproteomic Analysis of the Response of a Freshwater Microbial Community under Nutrient Enrichment

**DOI:** 10.3389/fmicb.2016.01172

**Published:** 2016-08-03

**Authors:** David A. Russo, Narciso Couto, Andrew P. Beckerman, Jagroop Pandhal

**Affiliations:** ^1^Department of Chemical and Biological Engineering, University of SheffieldSheffield, UK; ^2^Department of Animal and Plant Sciences, University of SheffieldSheffield, UK

**Keywords:** oligotrophic, eutrophic, metaproteomics, microbial loop, algae, freshwater

## Abstract

Eutrophication can lead to an uncontrollable increase in algal biomass, which has repercussions for the entire microbial and pelagic community. Studies have shown how nutrient enrichment affects microbial species succession, however details regarding the impact on community functionality are rare. Here, we applied a metaproteomic approach to investigate the functional changes to algal and bacterial communities, over time, in oligotrophic and eutrophic conditions, in freshwater microcosms. Samples were taken early during algal and cyanobacterial dominance and later under bacterial dominance. 1048 proteins, from the two treatments and two timepoints, were identified and quantified by their exponentially modified protein abundance index. In oligotrophic conditions, Bacteroidetes express extracellular hydrolases and Ton-B dependent receptors to degrade and transport high molecular weight compounds captured while attached to the phycosphere. Alpha- and Beta-proteobacteria were found to capture different substrates from algal exudate (carbohydrates and amino acids, respectively) suggesting resource partitioning to avoid direct competition. In eutrophic conditions, environmental adaptation proteins from cyanobacteria suggested better resilience compared to algae in a low carbon nutrient enriched environment. This study provides insight into differences in functional microbial processes between oligo- and eutrophic conditions at different timepoints and highlights how primary producers control bacterial resources in freshwater environments. The data have been deposited to the ProteomeXchange with identifier PXD004592.

## Introduction

Freshwater ecosystems are subjected to nutrient enrichment on a local, regional, and global scale in a process known as eutrophication. Due to human activity, global aquatic fluxes of nitrogen and phosphorus have been amplified by 108 and 400%, respectively (Falkowski et al., 2000). These nutrient imbalances have led to a drastic increase in the occurrence of algal blooms, an event where photoautotrophic biomass may increase by several orders of magnitude (Elser et al., 2007). During a bloom, high amounts of organic carbon and nutrients are channeled through the bacterial community and made available for higher trophic levels in what is known as the microbial loop (Azam et al., 1983). The microbial loop plays a crucial role in the biogeochemical cycling of elements, such as carbon, phosphorus and nitrogen, as well as organic matter. It is ultimately responsible for a substantial fraction of aquatic nutrient and energy fluxes (Azam and Malfatti, 2007). Thus, a better understanding of how the microbial loop and associated algae respond to nutrient enrichment, can reveal important features of how ecosystem processes are affected by eutrophication.

The development and application of “omics” technologies has allowed for an unprecedented view of microbial dynamics and their role in driving ecosystem function, including biogeochemical cycling of elements and decomposition and remineralization of organic matter. One approach is to obtain and sequence DNA from the microbial community in order to provide access to the genetic diversity of a microbial community (metagenomics). However, the genetic diversity gives us an incomplete view of what role these genes have in community processes. In contrast, metaproteomics can relate the intrinsic metabolic function by linking proteins to specific microbial activities and to specific organisms. Metaproteomics can thus address the long-standing objective in environmental microbiology of linking the identity of organisms comprising diversity in a community to ecosystem function (Hettich et al., 2013).

In the last few years metaproteomics has had a growing influence in aquatic environmental microbiology. It has been used to address questions about diversity, functional redundancy and provision of ecosystem services including nutrient recycling and energy transfer. For example, in one of the metaproteomic pioneering studies Giovannoni et al. (2005) demonstrated the ubiquity of proteorhodopsin-mediated light-driven proton pumps in bacteria (Giovannoni et al., 2005). Later, a study by Sowell et al. (2011) was the first of its kind to demonstrate the importance of high affinity transporters for substrate acquisition in marine bacteria (Sowell et al., 2011). Although, most of the notable metaproteomic aquatic studies have focused on marine environments, the tool has also been used in freshwater environments to examine, for example, the functional metaproteomes from the meromictic lake ecosystem in Antarctica (Ng et al., 2010; Lauro et al., 2011) or the microbes in Cayuga and Oneida Lake, New York (Hanson et al., 2014). The application of metaproteomics in such studies have successfully provided details regarding the importance of bacteriochlorophyll in the adaptation to low light (Ng et al., 2010), the metabolic traits that aid life in cold oligotrophic environments (Lauro et al., 2011) and nutrient cycling, photosynthesis and electron transport in freshwater lakes (Hanson et al., 2014).

In this paper we report a comprehensive discovery-driven (Aebersold et al., 2000) metaproteomic analysis of a freshwater microbial community under differing nutrient regimes to elucidate the predominant metabolic processes in each conditions. We expect that, overall, bacterial growth and abundance will be higher in the oligotrophic treatment but certain algal-bacterial processes (e.g., metabolite exchange) can benefit the microalgal community. In the eutrophic treatment, where algae have a growth advantage, proteins related to photosynthesis and energy generation should be highly expressed while it is expected that bacteria express proteins that aid adaptation to low dissolved organic matter (DOM) environments (e.g., switch from heterotrophy to autotrophy).

We inoculated microcosms with a microbial community subjected to two nutrient treatments to mimic oligotrophic and eutrophic conditions in freshwater lakes. Microcosms, as experimental systems, provide evidence for or against hypotheses that are difficult to test in nature (Drake and Kramer, 2011) and, here, allowed us to focus on the effects of nutrient enrichment on the microbial community. Bacterial, cyanobacteria and algal abundances were quantified throughout the experiment as were physicochemical measurements. The microbial metaproteome was extracted from two nutrient treatments (oligotrophic and eutrophic) at two time points. The time points were selected to represent phases of algal/cyanobacterial dominance and, later, heterotrophic bacterial dominance. For each treatment the extracted proteome was analyzed by nano-liquid chromatography–tandem mass spectrometry (LC-MS/MS). A meta-genetic community analysis of prokaryotic and eukaryotic diversity within the inoculum was used to generate a refined protein database for identifying proteins at the specified time-points. This approach reduced the spectral search space and led to reliable false discovery rate (FDR) statistics (Jagtap et al., 2013). The identified proteins at the two time points were then grouped into taxonomic and functional categories to link identity with function (Pandhal et al., 2008). We analyzed changes in protein expression in individual phylogenetic groups, over time and in both nutrient concentrations, to give an insight into the functional attributes of the major microbial players in the experimental microcosm community.

## Materials and Methods

### Microcosm Setup

We constructed replicate experimental biological communities in 30 L white, opaque, polypropylene vessels, 42 cm high and with an internal diameter of 31 cm. The microcosms were housed in controlled environment facilities at the Arthur Willis Environmental Centre at the University of Sheffield, UK. These were filled with 15 L of oligotrophic artificial freshwater growth medium (for detailed composition see **Supplementary Table S1**). Over the course of the experiment the microcosms were kept at constant temperature, 23°C, under 100 μmol m^-2^ s^-1^, provided by Hellelamp 400 watt IR Lamps HPS (Helle International, Ltd, UK), and 12:12 light dark cycle. A microbial community was introduced into each microcosm (detailed composition in **Supplementary Tables S2** and **S3**). This inoculum was sourced from 100 L of water samples collected at Weston Park Lake, Sheffield, UK (53°22′56.849″ N, 1°29′21.235″ W). The inoculum was filtered with a fine mesh cloth (maximum pore size 200 μm) to exclude big particles, protists and grazer populations (Downing et al., 1999). The filtered sample was cultured for 5 days in the conditions described to allow acclimation to the controlled conditions. Subsequently, each 15 L microcosm was inoculated with 2.5 L of this sample.

**FIGURE 1:**
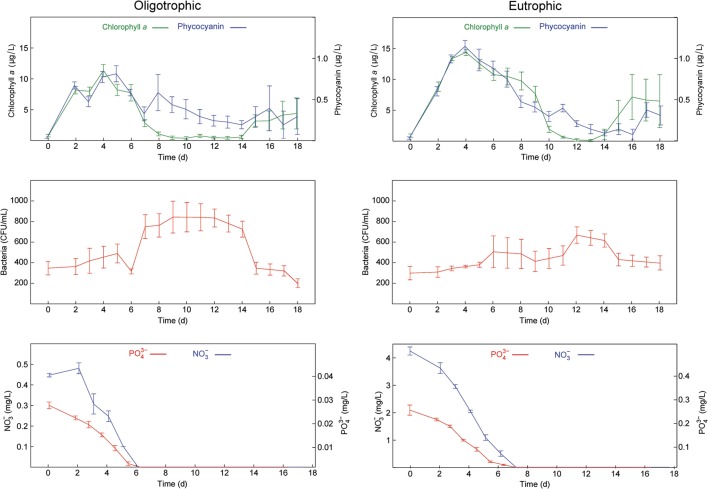
**Time series of the measured variables in the oligotrophic and eutrophic treatments.** **(Top)** Chlorophyll *a* and phycocyanin fluorescence. **(Middle)** Culturable heterotrophic bacteria. **(Bottom)** NO_3_^-^ and PO_4_^3-^. Error bars show standard errors (*n* = 3).

The inoculated microcosms were subjected to two nutrient treatments to mimic oligotrophic and eutrophic conditions in freshwater lakes. Our experimental elevation of initial nutrient levels followed United States Environmental Protection Agency guidelines for oligotrophic and eutrophic conditions in freshwater lakes and reservoirs (USEPA, 1986): (1) non-enriched growth medium to simulate oligotrophic conditions (NO_3_^-^ = 0.42 mg L^-1^ and PO_4_^3-^ = 0.03 mg L^-1^) and (2) NO_3_^-^ and PO_4_^3-^ enriched growth medium (NO_3_^-^ = 4.20 mg L^-1^ and PO_4_^3-^ = 0.31 mg L^-1^) to simulate eutrophic conditions. Each treatment was replicated 18 times, allowing for serial but replicated (*n* = 3 biological replicate microcosms) destructive sampling during the experiment. The experiment was run for 18 days to allow the added NO_3_^-^ and PO_4_^3-^ to deplete and generate batch microbial growth curves (see **Figure 1**). We also followed three control microcosms comprised of non-enriched growth medium, with no biological inoculum, allowing us to follow physicochemical variation in the absence of introduced biological activity (see **Supplementary Figure S1**).

### Sampling of Abiotic Variables

Over the course of the experiment dissolved oxygen (DO), pH, temperature, nitrate (NO_3_^-^) and phosphate (PO_4_^3-^) were monitored in order to link the abiotic variation to the changes observed in the biological variables. DO, pH, and temperature were measured at 12:00 and 18:00 daily with a Professional Plus Quatro (YSI, USA). 15 mL aliquots were collected and filtered (0.45 μm), daily, for the estimation of NO_3_^-^ and PO_4_^3-^ concentrations. NO_3_^-^ was estimated with a Dionex ICS-3000 ion chromatograph (Thermo Fisher Scientific, USA) using an AG18 2 mm × 250 mm column with a 0.25 mL min^-1^ flow rate and 31.04 mM potassium hydroxide as eluent. PO_4_^3-^ concentrations were measured according to protocols defined by British standards (BS EN ISO 6878:2004; BSI, 2004).

### Sampling of Biotic Variables

To estimate microalgae and cyanobacterial abundance, fluorescence was measured daily, at 12:00, with the AlgaeTorch (bbe Moldaenke GmbH, Germany). By measuring fluorescence, at 470, 525, and 610 nm for chlorophyll *a* and phycocyanin, the two spectral groups of microalgae and cyanobacteria, can be differentiated *in situ*. The relative amount of each group, expressed in terms of the equivalent amount of biomass per liter of water, was calculated according to Beutler et al. (2002).

Culturable heterotrophic bacteria were enumerated as an estimation of total bacteria (Lehman et al., 2001; Eaton and Franson, 2005; CSLC, 2009; Perkins et al., 2014) every 3 days by sampling 100 μL aliquots, in triplicate, plating on R2A agar (Oxoid, UK), incubating for 24 h at 37°C and counting colony forming units (CFU per mL). CFU were calculated with OpenCFU software (Geissmann, 2013). Because bacteria were only enumerated every 3 days, we used linear interpolation to generate a daily time series to obtain a uniform sample size across all variables. Interpolated values were calculated using the formula:

$$y=y_{1}+\left(y_{2}-y_{1}\right)\frac{x-x_{1}}{x_{2}-x_{1}}$$

where *y* is the missing value, *x* is the missing time point, *y*_1_, *y*_2_ are the two closest measured bacterial counts and *x*_1_, *x*_2_ are the respective time points.

### Protein Preparation

Microcosm samples were concentrated, in triplicate, at days 3 and 12 of the time course using a Centramate tangential flow filtration (TFF) system fitted with three 0.1 μm pore size Supor TFF membranes (Pall Corporation, USA). After every use, the filter system was sanitized with a 0.5 M sodium hydroxide solution and flushed with deionized water. The permeate was then filtered with a 3 μm pore size polycarbonate isopore membrane (EMD Millipore, USA) in order to obtain fractions dominated by free-living bacteria (<3 μm in size) and algae/particle-associated bacteria (>3 μm in size; Teeling et al., 2012). These fractions were harvested at 10, 000 × *g* for 15 min at 4°C. The resulting cell pellets were further washed in 0.5 M triethylammonium bicarbonate buffer (TEAB) prior to storage at -20°C. Cells were defrosted and resuspended in extraction buffer [250 μL of 0.5 M TEAB, 0.1% sodium dodecyl sulfate (SDS)] and 1 μL of halt protease inhibitor cocktail (Fisher Scientific, USA) incorporating a sonication bath step for 5 min with ice. The resulting suspension was submitted to five freeze-thaw cycles (each cycle corresponds to 2 min in liquid nitrogen and 5 min in a 37°C water bath; Ogunseitan, 1993). The lysed sample was centrifuged at 15,000 × *g* for 10 min at 4°C and the supernatant was transferred to a LoBind microcentrifuge tube (Eppendorf, Germany). The remaining cell pellet was resuspended in extraction buffer (125 μL) and homogenized with glass beads (425–600 μm) for 10 cycles (each cycle corresponds to 2 min homogenization and 2 min on ice). The lysed sample was centrifuged at 15,000 × *g* for 10 min at 4°C and the supernatants from both extraction methods were combined. 1 μL of benzonase nuclease (Sigma-Aldrich, USA) was added to the collected supernatants. Extracted proteins were precipitated overnight, at -20°C, using four volumes of acetone. The dried protein pellet was resuspended in 100 μL of 0.5 M TEAB and quantified using the 230/260 spectrophotometric assay described by Kalb and Bernlohr (1977). Biological replicates were pooled before reduction, alkylation, and digestion. This approach has been shown to be potentially valuable for proteomics studies where low amount of protein does not allow replication (Diz et al., 2009) whilst enhancing the opportunity to identify lower abundance proteins. Moreover, the small variances observed between replicate microcosms in terms of all biological and physiochemical measurements conducted (**Figure 1**; **Supplementary Figure S1**) gave further confidence to this approach. Protein samples (200 μg) were reduced with 20 mM tris-(2-carboxyethyl)-phosphine, at 60°C for 30 min, followed by alkylation with 10 mM iodoacetamide for 30 min in the dark. Samples were digested overnight, at 37°C, using trypsin (Promega, UK) 1:40 (trypsin to protein ratio) resuspended in 1 mM HCl. The samples were dried using a vacuum concentrator and stored at -20°C prior to fractionation.

### Chromatography and Mass Spectrometry

The first dimensional chromatographic separation, off-line, was performed on a Hypercarb porous graphitic column (particle size: 3 μm, length: 50 mm, diameter: 2.1 mm, pore size: 5 μm; Thermo-Dionex, USA) on an Ultimate 3000 UHPLC (Thermo-Dionex, USA). Peptides were resuspended in 200 μL of Buffer A [0.1% (v/v) trifluoroacetic acid (TFA) and 3% (v/v) HPLC-grade acetonitrile (ACN) in HPLC-grade water] and eluted using a linear gradient of Buffer B [0.1% (v/v) TFA and 97% (v/v) ACN in HPLC-grade water] ranging from 5 to 60% over 120 min with a flow rate of 0.2 mL min^-1^. Peptide elution was monitored at a wavelength of 214 nm and with Chromeleon software, version 6.8 (Thermo-Dionex, USA). Fractions were collected every 2 min, between 10 and 120 min, using a Foxy Junior (Teledyne Isco, USA) fraction collector and dried using a vacuum concentrator. Dried fractions were stored at -20°C prior to mass spectrometry analysis. The second dimensional chromatographic separation of each peptide fraction was performed on a nano-LC-CSI-MS/MS system. In this system a U3000 RSLCnano LC (Thermo-Dionex, USA), containing a trap column (300 μm × 5 mm packed with PepMap C18, 5 μm, 100 Å wide pore, Dionex) followed by a reverse phase nano-column (75 μm × 150 mm packed with PepMap C18, 2 μm, 100 Å wide pore, Dionex), was coupled to an ultra-high resolution quadrupole time-of-flight (UHR maXis Q-ToF 3G) mass spectrometer (Bruker, Germany) equipped with an Advance CaptiveSpray ion source. Peptide fractions were resuspended in loading buffer [0.1% (v/v) TFA and 3% (v/v) ACN in HPLC-grade water] and two injections were made. A 90 min linear gradient elution was performed using buffer A [0.1% (v/v) formic acid (FA) and 3% (v/v) ACN in HPLC-grade water] and buffer B [0.1% (v/v) FA and 97% (v/v) ACN in HPLC-grade water], during which buffer B increased from 4 to 40% at a flow rate of 0.3 μL min^-1^. On the mass spectrometer, the following settings were specified: endplate Offset -500 V, capillary voltage 1000 V, nebulizer gas 0.4 bar, dry gas 6.0 L min^-1^, and dry temperature 150°C. Mass range: 50–2200 *m/z*, at 4 Hz. Lock mass was used for enabling mass acquisition correction in real time, therefore high mass accuracy data were obtained. Data were acquired for positive ions in a dependent acquisition mode with the three most intense double, triple or quadruple charges species selected for further analysis by tandem mass spectrometry (MS/MS) under collision induced dissociation (CID) conditions where nitrogen was used as collision gas.

### 16 and 18S rDNA Gene Sequencing of Inoculum

#### DNA Extraction

Inoculum samples were lysed in 50 mM Tris-HCl (pH 8.0), 10 mM EDTA and 10% (w/v) SDS by vortexing with glass beads. DNA was extracted with a standard phenol-chloroform extraction protocol (Sambrook and Russel, 2001). The DNA was precipitated using sodium acetate (50 μL of 3 M stock solution, pH 4.8–5.2) and ice-cold ethanol. PCR amplification, product pooling, purification sequencing and bioinformatics and statistical analysis were performed by Research and Testing Laboratory (Lubbock, TX, USA).

#### PCR Amplification

Markers were amplified from DNA extractions using adapted Illumina tagged primers. Forward primers were constructed with Illumina adapter i5 (AATGATACGGCGACCACCGAGATCTACAC) an 8–10 bp barcode, a primer pad and either primer 28F (GAGTTTGATCNTGGCTCAG) or TAReukF (CCAGCASCYGCGGTAATTCC). Reverse primers were constructed with Illumina adapter i7 (CAAGCAGAAGACGGCATACGAGAT) an 8–10 bp barcode, a primer pad and either primer 519R (GTNTTACNGCGGCKGCTG) or TAReukR (ACTTTCGTTCTTGATYRA). Primer pads were used to ensure a primer melting temperature of 63–66°C, as per the Schloss method (Schloss et al., 2009). Reactions were performed using corresponding primer pairs (i.e., 28F × 519R and TAReukF × TAReukR) using the Qiagen HotStar Taq master mix (Qiagen, Inc., Valencia, CA, USA) adding 1 uL of each 5 uM primer, and 1 uL of template to make a final 25 μL reaction volume, with a thermal cycling profile of 95°C for 5 min., then 35 cycles of 94°C for 30 s, 54°C for 40 s, 72°C for 1 min, followed by one cycle of 72°C for 10 min. Amplified products were visualized with eGels (Life Technologies, Grand Island, NY, USA) and pooled. Pools were purified (size selected) through two rounds of 0.7x Agencourt AMPure XP (BeckmanCoulter, Indianapolis, IN, USA) as per manufacturer’s instructions, before quantification with a Quibit 2.0 fluorometer (Life Technologies). Finally pools were loaded and sequenced on an Illumina MiSeq (Illumina, Inc., San Diego, CA, USA) 2 × 300 flow cell at 10 pM. The sequence data are available from the European Nucleotide Archive under Study Accession Number PRJEB12443, and Sample Accession Numbers ERS1037123 (16S DNA) and ERS1037124 (18S DNA).

#### Bioinformatic and Statistical Analysis

Initially the forward and reverse reads were taken and merged together using the PEAR Illumina paired-end read merger (Zhang et al., 2014). Reads were then filtered for quality by trimming them once average quality dropped below 25 and prefix dereplication was performed using the USEARCH algorithm (Edgar, 2010). Sequences below 100 bp were not written to the output file and no minimum cluster size restriction was applied. Clustering was performed at a 4% divergence using the USEARCH clustering algorithm (Edgar, 2010). Clusters containing less than two members were removed. OTU selection was performed using the UPARSE OTU selection algorithm (Edgar, 2013). Chimeras were then checked for and removed from the selected OTUs using the UCHIME chimera detection software executed in *de novo* mode (Edgar et al., 2011). Reads were then mapped to their corresponding non-chimeric cluster using the USEARCH global alignment algorithm (Edgar, 2010). The denoised sequences were demultiplexed and the primer sequences removed. These sequences were then clustered into OTUs using the UPARSE algorithm (Edgar, 2013) which assigns each of the original reads back to their OTUs and writes the mapping data to an OTU table file. The centroid sequence from each OTU cluster was then run against the USEARCH global alignment algorithm and the taxonomic identification was done using a NCBI database as described in Bokulich et al. (2015). Finally, the OTU table output from sequence clustering was collated with the output generated during taxonomic identification and a new OTU table with the taxonomic information tied to each cluster was created (Bokulich et al., 2015).

### Protein Identification and Quantification

All MS and MS/MS raw spectra were processed using Data Analysis 4.1 software (Bruker, Germany) and the spectra from each Bruker analysis file were output as a mascot generic file (MGF) for subsequent database searches using Mascot Daemon (version 2.5.1, Matrix Science, USA). The peptide spectra were searched against a eukaryotic and a prokaryotic database created by collating all Uniprot entries (retrieved on 24 February 2015) from organisms with an abundance of >1% in the 16 and 18S rDNA survey of our inoculum (**Table 1**, full list in **Supplementary Tables S2** and **S3**). This search was undertaken utilizing the two-step approach described in Jagtap et al. (2013). Briefly, the initial database search was done without any FDR limitation and then was followed by a second search with a 1% FDR threshold against a refined database created by extracting the protein identifications derived from the first search. FDRs for assigning a peptide match were determined from the ratio of the number of peptides that matched to the reversed sequence eukaryotic and prokaryotic databases to the number of peptides matched to the same databases in the forward sequence direction. The following search parameters were applied to both searches: up to one missed cleavage with trypsin, fixed modification of cysteine residues by carbamidomethylation, variable modification of methionine by oxidation, instrument specification ESI Q-ToF, peptide charge: 2+, 3+ and 4+, precursor mass tolerance of ±0.2 Da and fragment-ion mass tolerance of ±0.02 Da. For the second search only matches above a 95% confidence homology threshold, with significant scores defined by Mascot probability analysis, and a 1% FDR cut-off were considered confidently matched peptides. ‘Show sub-sets’ and ‘require bold red’ were applied on initial Mascot results to eliminate redundancy. The highest score for a given peptide mass (best match to that predicted in the database) was used to identify proteins, which in turn were assigned a most probable host. Furthermore, only when two or more unique peptides, per protein, were matched did we consider a protein identified. Protein abundance was relatively estimated through the exponentially modified protein abundance index (emPAI; Ishihama et al., 2005). emPAI is an approximate, label-free, relative quantitation of the proteins. This method is based on the protein abundance index (PAI) that calculates the number of different observed peptides divided by the number of observable peptides as a measure of abundance. This PAI value is then exponentially modified to derive the emPAI score. A protein abundance is then finally calculated after normalizing the emPAI score for a protein by dividing it by the sum of the emPAI scores for all identified proteins (Ishihama et al., 2005). The mass spectrometry proteomics data have been deposited to the ProteomeXchange Consortium^1^ via the PRIDE partner repository (Vizcaíno et al., 2013) with the dataset identifier PXD004592 and DOI 10.6019/PXD004592.

**Table 1:** List of the eukaryotic and prokaryotic organisms in the experimental freshwater microbial community inoculum, with an abundance higher than 1%, as determined by 16 and 18S rDNA sequencing.

Eukaryotic organisms	%	Prokaryotic organisms	%
*Chloromonas pseudoplatyrhyncha*	26.93	*Rhodoferax* sp.	21.94
*Stephanodiscus* sp.	18.17	Unsequenced organisms	17.84
Unsequenced organisms	17.87	*Flavobacterium* sp.	9.43
*Chromulinaceae* sp.	8.48	*Anabaena* sp.	8.85
*Synedra angustissima*	4.99	*Brevundimonas diminuta*	4.20
*Ochromonadales* sp.	3.01	*Hydrogenophaga* sp.	3.41
*Chlamydomonas* sp.	2.98	*Runella limosa*	2.47
*Micractinium pusillum*	1.62	*Haliscomenobacter* sp.	2.43
*Chlorella* sp.	1.08	*Rhodobacter* sp.	2.34
*Pythiaceae* sp.	1.07	*Planktophila limnetica*	2.13
		*Agrobacterium tumefaciens*	2.11
		*Sphingobacterium* sp.	2.03
		*Ochrobactrum tritici*	1.98
		*Brevundimonas variabilis*	1.83
		*Sphingomonas* sp.	1.73
		*Curvibacter* sp.	1.48
		*Phenylobacterium falsum*	1.42
		*Roseomonas stagni*	1.24
		*Oceanicaulis* sp.	1.03


*These organisms were used to guide creation of a protein database.*

### Functional Classification of Proteins

Proteins were semi-automatically attributed a functional classification. Briefly, a list of UniProt accession numbers was collated from each sample and queried utilizing the UniProt Retrieve/ID mapping tool^2^ Column options ‘Keywords’ and ‘Gene ontology (biological process)’ were selected. Incomplete or ambiguous annotations were then manually completed by searching for the individual UniProt accession numbers on Pfam^3^ and EggNOG^4^

## Results and Discussion

### Biological and Physicochemical Measurements

The time points chosen for metaproteomic analysis of our samples were based on biological and physicochemical variables measured in our microcosms. Algal and cyanobacterial abundance peaked at day 3, and was maintained until nitrate and phosphate concentrations were no longer in detectable range, but declined after their depletion between days 6 and 8 (**Figure 1**). The decline in abundance of algae and cyanobacteria was followed by a peak of bacterial abundance at day 12 (**Figure 1**). Heterotrophic bacterial growth is known to be stimulated by an accumulation of DOM derived from senescent algae and cyanobacteria. Hence, in a given body of water the peak of heterotrophic bacterial activity tends to follow the peak of primary production.

Based on these patterns the samples selected for metaproteomic analyses were harvested at day 3, the peak of algal and cyanobacterial concentrations (early oligo- and eutrophic) and day 12, the peak of bacterial concentrations (late oligo- and eutrophic). The comparative analysis of these biologically distinct time points can provide information regarding the activity of the microbial community during algal/cyanobacterial dominance and bacterial dominance under low and high nutrient conditions.

Similar patterns were observed in DO, pH, and temperature measurements in both nutrient treatments (**Supplementary Figure S1**) and together with the low level of variation observed in biological measurements (**Figure 1**), provided additional confidence in the sample pooling approach for metaproteomics analyses.

### Metaproteomic Database Creation and Search Results

The 18S rDNA sequencing of the microcosm inoculum indicated that, at day 0, the eukaryotic community was predominantly composed of Chlorophyceae (e.g., Chloromonas), Bacillariophyceae (e.g., Stephanodiscus), and Chrysophyceae (e.g., Chromulinaceae). These are typical unicellular freshwater microalgal species that are normally found in freshwater oligotrophic environments (Bailey-Watts, 1992).

The 16S rDNA sequencing of the microcosm inoculum showed that, at day 0, the prokaryotic community was predominantly composed of Alpha-proteobacteria (e.g., Brevundimonas), Beta-proteobacteria (e.g., Rhodoferax), Flaviobacteria (e.g., Flaviobacterium), and Cyanophyceae (e.g., *Anabaena*). Proteobacteria and Flaviobacteria are ubiquitous in freshwater environments with the latter being known to dominate eutrophic environments where phytoplankton population numbers are high (Eiler and Bertilsson, 2007; Newton et al., 2011). *Anabaena* is a well-researched freshwater cyanobacterium that is known to occasionally be responsible for harmful algal blooms (Elser et al., 2007).

This list of organisms was utilized to create a eukaryotic and a prokaryotic protein database by collating all Uniprot entries from organisms with an abundance of >1% in the 16 and 18S rDNA survey of the inoculum (**Table 1**; full list in **Supplementary Tables S2** and **S3**). This approach was applied to limit the size of the resulting protein databases, which can lead to high false positive rates, and also in accordance with the nature of mass spectrometry based proteomics, where only the most abundant proteins are identified. As a result the eukaryotic and prokaryotic databases contained 86336 and 350356 sequence entries, respectively. These databases were utilized to identify proteins from peptide fragments in a two-step approach (Jagtap et al., 2013). This approach is valuable when dealing with large metaproteomic database searches where the target and decoy identifications may overlap significantly and valuable identifications are missed out (Muth et al., 2015). Proteins of eight samples, representing the two time points selected under different nutrient concentrations (early and late oligo- and eutrophic) and two size separated fractions, [free-living bacteria (<3 μm in size) and algae/particle-associated bacteria (>3 μm in size)] were identified and an average of 131 ± 28 proteins, above a 95% confidence homology threshold, a 1% FDR cut-off and with two unique peptides, were identified per sample. Values were pooled by broad protein annotation and taxonomic categories to evaluate differences between early and late oligotrophic and eutrophic conditions. The average coefficient of variation (CV) of emPAI across three biological replicates in non-fractionated protein samples, was 0.15. The average relative variance (RV) was also determined logarithmically and was 0.82 indicating a 18% discrepancy for relative quantitation. This provided us with confidence of a 1.5 fold cut off to minimize the identification of false positive differentially regulated proteins.

### Phylogenetic Diversity According to the Metaproteomic Spectra

Identifying discrepancies between the phylogenetic classification of the identified proteins and the 16 and 18S rDNA sequencing used to create the metaproteomic database can indicate if any specific phylogenetic group is inadequately represented. rDNA sequencing was performed on the inoculum (i.e., at day 0 of the experiment) and therefore a direct comparison with the metaproteomes is not possible. Nevertheless, the 16 and 18S rDNA sequencing information provided a template to which the metaproteome could be compared. Of the total number of identified proteins in the >3 μm fraction, across all four samples, 48–55% were identified in Chlorophyta, 9–27% in Heterokontophyta and, finally, 12–33% in Cyanobacteria.

A more detailed look at the genus level of the phylogenetic distribution showed that *Chlamydomonas* sp. proteins are most abundant in the early part of the time series [oligotrophic (50%) and eutrophic (43%)], *Chlorella* sp. in late oligotrophic (39%) and *Anabaena* sp. in late eutrophic (37%) conditions. The 18S rDNA sequencing indicated that the abundance of the *Chlorella* genus was only 1.08% of the initial inoculum. However, proteins belonging to the *Chlorella* genus represented up to 39% of total protein. This is most likely due to an over representation of *Chlorella* sp. in the metaproteomic database due to it being a model genus with a large number of sequences available in Uniprot.

The phylogenetic distribution, based on proteins identified across the samples, mostly fitted with the biological measurements. Microalgae concentrations were always higher than cyanobacteria concentrations over the course of the experiment (**Figure 1**). Cyanobacteria had the highest number of proteins identified in late eutrophic (37%) mostly due to the expression of highly abundant proteins related to carbon concentration mechanisms (CCMs). It has been suggested that this is a mechanism of survival under adverse conditions that could, in the long term, favor cyanobacterial populations (Yeates et al., 2008).

Of the total number of identified proteins in the <3 μm fraction, across all four samples, 60–73% were identified in Proteobacteria and 27–40% in Bacteroidetes. Bacteroidetes proteins were more abundant in early oligotrophic conditions whereas Proteobacteria were more abundant in late oligotrophic and early eutrophic conditions. A more detailed look at the class level of the phylogenetic distribution showed that Bacteroidetes proteins were more abundant in the early phase [oligotrophic (29%) and eutrophic (30%)] while Alpha-proteobacteria proteins were abundant in late oligotrophic (30%) and Beta-proteobacteria proteins in late eutrophic (30%) conditions.

Again, the taxonomic community composition found by 16S rDNA sequencing and the metaproteome were in agreement and the phylogenetic distribution across the samples supports previous observations of these organisms. Bacteroidetes typically establish mutualistic relationships with algae on the cell surface and are more abundant when algal concentrations are high such as earlier in the time series (**Figure 1**). Alpha-proteobacteria and Beta-proteobacteria, as opportunistic heterotrophs, therefore thrive in the presence of DOM derived from algal and cyanobacterial decay which was abundant later in the time series (**Figure 1**; Teeling et al., 2012).

### Functional Classification of Proteins

The distribution of identified proteins by their functional classification resulted in 20 distinct functional categories. The grouping of proteins identified in each fraction and nutrient condition can give an overview of how the community function differed over time and nutrient enrichment.

**FIGURE 2:**
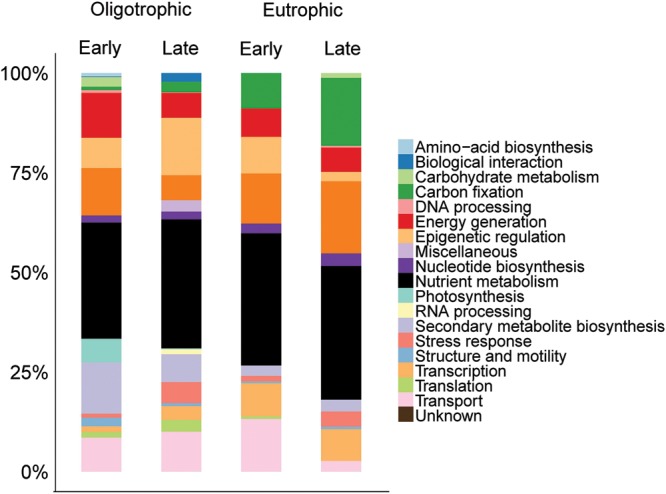
**Comparison across samples of the distribution of identified proteins by their functional classification in the >3 μm fraction**.

Of the total number of identified proteins, 25% were involved in photosynthesis, thus, dominating the >3 μm fraction (**Figure 2**). 9% of the total protein library were classified with unknown function. Proteins with assigned functions in each individual samples were dominated by photosynthesis (early oligotrophic, 21%; late oligotrophic, 25%; early eutrophic, 26%; late eutrophic, 30%). On the individual protein level, photosystem II (PSII) CP43 reaction center proteins were the most abundant in early oligotrophic (8%), histone H2 proteins in late oligotrophic (14%), PSII CP43 reaction center proteins and histone H4 proteins (8% each) in early eutrophic and microcompartment proteins (16%) in late eutrophic conditions.

In agreement with our findings, Hanson et al. (2014) observed that in both freshwater and marine surface samples (i.e., rich in primary production) there was widespread evidence of photosynthesis (e.g., PSII) and carbon fixation [e.g., ribulose-1,5-bisphosphate carboxylase oxygenase (RuBisCO; EC 4.1.1.39)]. Although our samples were not rich in RuBisCO, the presence of microcompartment proteins are evidence of carbon fixation.

**FIGURE 3:**
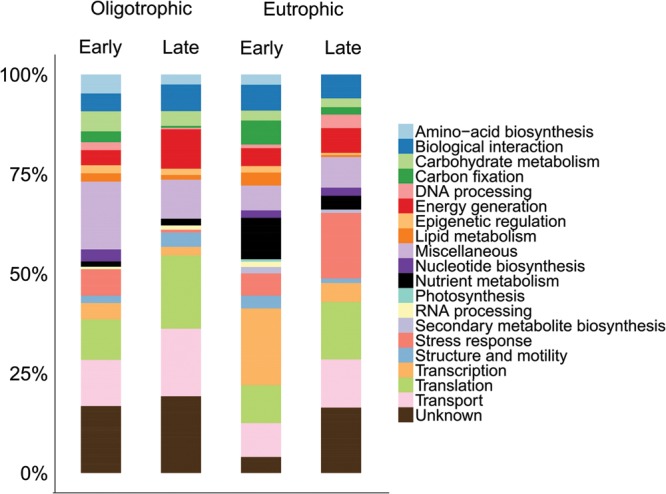
**Comparison across samples of the distribution of identified proteins by their functional classification in the <3 μm fraction**.

Of the total number of identified proteins, transport (12%) and translation (12%) proteins were predominant in the <3 μm fraction (**Figure 3**). A more detailed view showed early oligotrophic conditions dominated by transport proteins (12%), late oligotrophic by translation proteins (18%), early eutrophic by transcription proteins (19%) and late eutrophic by stress response proteins (16%). On the individual protein level the ATP-binding cassette (ABC) transporter proteins were the most abundant in early oligotrophic (5%), elongation factor proteins in late oligotrophic (16%), DNA-directed RNA polymerase subunit beta (EC 2.7.7.6) in early eutrophic (19%) and ABC transporter proteins (7%) in late eutrophic conditions. Proteins involved in transport (e.g., ABC transporters), translation (e.g., elongation factors) and transcription (DNA-directed RNA polymerase subunit beta) are amongst the most commonly identified proteins in environmental samples (Ng et al., 2010; Sowell et al., 2011; Hanson et al., 2014).

### Metaproteomic Analysis of Microcosm Microbial Activity

**FIGURE 4:**
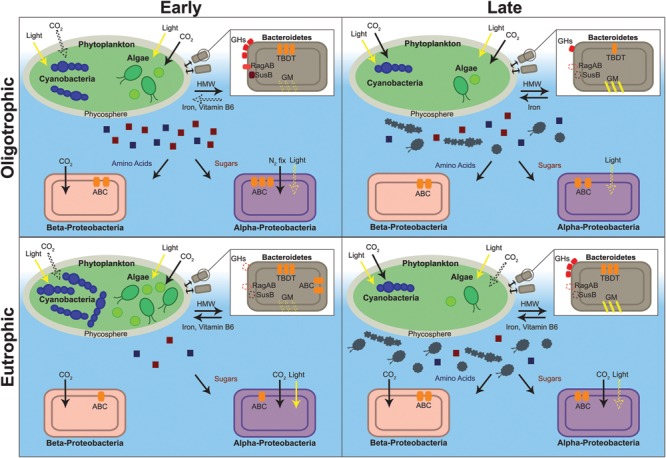
**Depiction of the metabolic characteristics of oligotrophic and eutrophic communities inferred from the metaproteome.** Red and blue squares depict algal and cyanobacterial exudate (red, sugars; blue, amino acids). Gray algae and cyanobacteria depict senescent cells. Structures and processes that are hypothesized to be present, albeit with no direct evidence from our dataset, are depicted with a dashed line. ABC, ATP-binding cassette transporter; GHs, glycoside hydrolases; GM, gliding motility; HMW, high molecular weight compounds; N_2_ fix, nitrogen fixation; TBDT, Ton-B-dependent transporter.

Having identified protein functional groups in eukaryotic and prokaryotic organisms throughout our samples, we can now assess functional differences between oligotrophic and eutrophic conditions, early and late in the time series. We found several patterns previously documented and several unexpected differences between time points and between oligotrophic and eutrophic conditions within each time point. **Figure 4** captures a summary of the functional differences among the times and treatments, and we now refer to this figure, and **Figures 2** and **3**, to provide detail.

First, virtually all the photosynthesis and carbon fixation proteins were identified in *Anabaena* sp., *Chlamydomonas* sp., and *Chlorella* sp. This is similar to previous metaproteomic studies where the freshwater surface is typically rich in photosynthetic organisms (Hanson et al., 2014). The most abundant of the two categories was photosynthesis (emPAI = 11.89) and it represented 40% of all proteins expressed by photoautotrophic organisms. The majority of the proteins were components of PSII (e.g., reaction center components). This was expected because PSII proteins are 40–90% more abundant than PSI proteins and are the most abundant membrane proteins in algae and cyanobacteria (Nobel, 2005). Photosynthetic proteins were abundant in both timepoints (early, emPAI = 5.27 and late, emPAI = 5.61) and in both nutrient treatments (oligotrophic, emPAI = 5.31 and eutrophic, emPAI = 5.57), suggesting that the phototrophs are demanding a constant energy supply, even outside of the exponential growth phase.

Second, amongst the photosynthetic microbes, there is interest in identifying mechanisms that could potentially favor cyanobacteria in eutrophic conditions. The increase in the number of nutrient enriched water bodies has led to issues with freshwater quality and the proliferation of harmful cyanobacteria (O’Neil et al., 2012). There have been numerous proteomics studies of toxic bloom causing cyanobacteria that have focused on the molecular mechanisms of pure cultures. For example, a study of the proteomes of six toxic and non-toxic strains of *Microcystis aeruginosa* linked nitrogen regulation to toxicity (Alexova et al., 2011) and another study, of *Anabaena* sp. Strain 90, linked phosphorus starvation to the down regulation of the Calvin cycle and amino-acid biosynthesis (Teikari et al., 2015). Studies such as these provide valuable information regarding species in isolation, however, metaproteomics can go a step further and contextualize these findings within the microbial community structure and dynamics.

Our microcosm data showed that pigment proteins in *Anabaena* sp. were less abundant in oligotrophic than in eutrophic conditions (oligotrophic, emPAI = 0.42; eutrophic, emPAI = 0.96). A similar pattern was found for cyanobacterial proteins with roles in carbon fixation (oligotrophic, emPAI = 0.14; eutrophic, emPAI = 2.03). Cyanobacteria have the ability to adapt to different environments by adjusting their light harvesting abilities (i.e., increase in pigments) and carbon fixation mechanisms. However, these adaptation processes can be hampered by insufficient nutrient supply (Tilzer, 1987). Grossman et al. (1993) showed that during nutrient starvation, there is a rapid degradation of the phycobilisome. Phycobilisome degradation can provide nutrient-starved cells with amino acids used for the synthesis of proteins important for their metabolism (Grossman et al., 1993). This suggests that nutrient enrichment would allow cyanobacteria to increase pigment numbers, thus increasing light harvesting ability, and outcompete algal species in eutrophic conditions (Tilzer, 1987).

Regarding carbon fixation, microcompartment proteins were identified in *Anabaena* sp. and were only found in in late eutrophic conditions (eutrophic, emPAI = 1.52). Microcompartments sequester specific proteins in prokaryotic cells and are involved in CCMs in low CO_2_ conditions. The carboxysome, a bacterial microcompartment that is found in cyanobacteria and some chemoautotrophs, encapsulates RuBisCO and carbonic anhydrase (EC 4.2.1.1) The carbonic anhydrase reversibly catalyzes the conversion of bicarbonate into carbon dioxide within the carboxysome therefore acting both as a intracellular equilibrator and a CO_2_ concentrating mechanism (Yeates et al., 2008). However, no carbonic anhydrases were identified in our dataset. A higher abundance of carbon fixation proteins in *Anabaena* sp., in eutrophic conditions, indicates that carbon requirement was higher, likely matching higher photosynthesis rates compared to the oligotrophic conditions, where low nitrogen and phosphorus concentrations are likely limiting factors and therefore, not allowing the population to reach a point of carbon limitation.

Finally, carbon fixation proteins in *Chlamydomonas* sp. were also more abundant in eutrophic conditions (oligotrophic, emPAI = 0.17; eutrophic, emPAI = 0.40). The proteins identified were mainly involved in the Calvin cycle (i.e., RuBisCO), however, unexpectedly, a low-CO_2_ inducible protein (LCIB) was identified. The LCIB is located around the pyrenoid and traps CO_2_, either from escaping from the pyrenoid or entering from outside the cell, into the stromal bicarbonate pool thus, functioning as a CCM (Wang and Spalding, 2014). Wang and Spalding hypothesized that this system may reflect a versatile regulatory mechanism present in eukaryotic algae for acclimating quickly to changes in CO_2_ availability that frequently occur in their natural environments. The possibility of switching between an energy-intensive bicarbonate transport system (low CO_2_) and diffusion based CO_2_ uptake system (high CO_2_) that may be energetically less costly, would enable faster growth at a lower energy cost.

These observations suggest that algae and cyanobacteria both adapt to carbon limitation through an increase in carbon fixation proteins and the deployment of CCMs (e.g., carboxysomes). In a low-carbon lake, the microbial population may thus fix atmospheric CO_2_ to correct the carbon deficiency and grow in proportion to existing nitrogen and phosphorus levels. This maps onto the hypothesis that carbon limitation may not be adequate for algal or cyanobacterial bloom mitigation (Schindler et al., 2008).

#### Bacterial Photosynthesis and Carbon Fixation

Heterotrophic bacteria are known to be responsible for the bulk of sequestration and remineralization of organic matter in phytoplankton associated bacterial assemblages (Buchan et al., 2014). However, the role of photoheterotrophic and chemoautotrophic bacteria in these assemblages, and how they vary along environmental gradients, remains under-studied (Yutin et al., 2007; Ng et al., 2010). The observations to date suggest that these bacteria are ubiquitous but have a preference for carbon limiting environments such as the DOM poor conditions found early in the time series, during algal and cyanobacterial dominance, in this study (**Figure 4**).

In support of this hypothesis, bacterial photosynthesis [i.e., magnesium chelatase (EC 6.6.1.1)] and carbon fixation proteins (i.e., RuBisCO, carbonic anhydrase) were identified in both treatments (**Figure 4**) with predominance early in the time series (early, emPAI = 1.28; late, emPAI = 0.11) and eutrophic conditions (oligotrophic, emPAI = 0.57; eutrophic, emPAI = 0.82). Specifically, in Alpha- and Beta-proteobacteria, magnesium chelatase (emPAI = 0.03), which is involved in bacteriochlorophyll biosynthesis, was identified in early oligotrophic (emPAI = 0.03) and RuBisCO was present in both nutrient treatments. Alpha- and Beta-proteobacteria include several mixotrophic species that are known to perform aerobic and anaerobic respiration and use combinations of photo-, chemo-, auto- and heterotrophic metabolism to adapt to different environmental conditions. Some of these bacterial species perform anoxygenic photosynthesis, where light energy is captured and converted to ATP without the production of oxygen, and are described as photo(chemo)heterotrophs due to their requirement of organic carbon. It has been suggested that these bacteria grow chemoheterotrophically but utilize light as an additional energy source (Eiler, 2006).

The low levels of DOM in early oligotrophic conditions (i.e., algal and cyanobacterial dominance) provided a niche for phototrophy and autotrophy. Later, in the presence of DOM derived from algal and cyanobacterial cell lysis, the bacterial groups changed to a heterotrophic metabolism. This suggests that an increase in Proteobacterial metabolism depends more on the concentrations of organic matter than on nitrogen and phosphorus, and that bacterial mixotrophy is ubiquitous in low DOM freshwater environments. This has consequences for biogeochemical models such as the microbial loop. The classic separation of primary and secondary producers into photoautotrophs and organoheterotrophs, respectively, is no longer valid and may lead to the underestimation of bacterial biomass production and their importance to higher trophic levels (Eiler, 2006).

Finally, other bacterial groups found in our study, such as the Bacteroidetes, can also use non-photosynthetic routes of light-dependent energy generation. Previous metaproteomic studies have shown that proteorhodopsin, a light driven proton pump, is ubiquitous in marine and freshwater environments (Atamna-Ismaeel et al., 2008; Williams et al., 2013). Its expression has been linked to survival in situations where sources of energy are limiting and cells have to resort to alternative means of generating energy (González et al., 2008). However, proteorhodopsin was not detected either because of non-expression in the conditions tested, low abundance or low solubility of the protein; proteorhodopsin contains seven transmembrane helices and is imbedded in the plasma membrane thus making it difficult to solubilize and detect (Sowell et al., 2009).

#### Bacteroidetes: An Algal Associated Bacterial Group

The Bacteroidetes phylum has been hypothesized to specialize in degrading high molecular weight (HMW) compounds and growing whilst attached to particles, surfaces, and algal cells (Teeling et al., 2012; Fernandez-Gomez et al., 2013; Williams et al., 2013). Teeling et al. (2012) also observed that the bacterial response to a coastal algal bloom was characterized by an initial surge in Bacteroidetes abundance. Thus, it was hypothesized that this group colonizes the phytoplankton surface and acts as “first responders” to algal blooms (Williams et al., 2013). Therefore, the identification of proteins that suggest a tight algae – bacteria relationship were expected to be found early in the time series. Also, the higher algal concentrations in eutrophic conditions (**Figure 1**) would presumably provide a richer environment for the Bacteroidetes population.

As predicted, in both oligotrophic and eutrophic treatments, Bacteroidetes proteins were considerably more abundant in the early phase of the experiment (early, emPAI = 14.84, late, emPAI = 5.85) with several of the identified proteins suggesting a close association with algae (**Figure 4**). First, several proteins attributed to the TonB-dependent transporter (TBDT) system were identified. TBDTs are involved in proton motive force-dependent outer membrane transport and once thought to be restricted to iron-chelating compounds (i.e., siderophores) and vitamin B12 uptake. Recently TBDTs have been found to specialize in the uptake of HMW compounds that are too large to diffuse via porins (e.g., polysaccharides, proteins; Blanvillain et al., 2007). In Bacteroidetes, the genes for the TBDT system are located in the same gene cluster as several of the polymer capture (e.g., starch utilization system) and degradation genes [e.g., glycoside hydrolases (GHs), peptidases] suggesting an integrated regulation of capture, degradation, and transport of complex substrates (Fernandez-Gomez et al., 2013). The proteins identified in our Bacteroidetes dataset support this suggestion.

Second, three starch utilization system proteins (SusD/RagB) in Bacteroidetes were identified early in the time series (**Figure 4**). SusD proteins are present at the surface of the cell and they mediate starch-binding before transport into the periplasm for degradation. RagAB is involved in binding exogenous proteins (Gilbert, 2008; Dong et al., 2014). GHs from several families (GH3, GH29, GH30, and GH92), together with three peptidases [methionine aminopeptidase (EC 3.4.11.18), peptidase M16, peptidyl-dipeptidase (EC 3.4.15.1)] were also identified. As mentioned previously GHs are carbohydrate-active enzymes (CAZymes) specialized in the uptake and breakdown of complex carbohydrates, especially algal polysaccharides (Teeling et al., 2012; Mann et al., 2013). Together with peptidases these enzymes are responsible for extracellular breakdown of organic matter in order to be transported into the cytoplasm by the TBDT system.

Finally, the identification of proteins with cell adhesion functions (intimin, thrombospondin 1, gliding motility protein and YD repeat) provides further evidence that this bacterial phylum specializes in surface attachment. Intimin, thrombospondin and YD repeat protein are adhesive proteins that mediate cell-to-cell interactions and gliding mobility proteins allow exploration of solid surfaces (McBride, 2001). Other bacterial species utilize gliding motility for essential life cycle processes (e.g., swarming, predation) usually in coordinated groups but also as isolated adventurous individuals (Nan and Zusman, 2011). In a similar way Bacteroidetes species may use gliding motility to follow algal exudate trails and to move to advantageous positions within the phycosphere, the microscale mucus region rich in organic matter that surrounds algal and cyanobacterial cells. This could confer a competitive advantage over free-floating bacterial species.

When contrasting oligo- and eutrophic treatments, Bacteroidetes associated proteins were, unexpectedly, more abundant in oligotrophic rather than eutrophic conditions (oligotrophic, emPAI = 14.02; eutrophic, emPAI = 6.67). In eutrophic conditions proteins attributed to transport, macromolecule degradation, outer membrane capture and chemotaxis were virtually non-existent (**Figure 4**). The fact that very little capture and degradation was occurring in eutrophic conditions suggests algal exudation was substantially lower. In the past, it has been hypothesized that nutrient limitation is a requirement for algal and cyanobacterial exudation (Wood and Van Valen, 1990; Guenet et al., 2010). Van den Meersche et al. (2004) determined that contribution of algal derived DOM to the experimental ecosystem carbon pool varied from ∼2% (nutrient-replete early bloom) to 65% (nutrient-deplete mid-late bloom). Thus, the stimulation of DOM release, by nutrient limiting conditions, paradoxically provides carbon substrates for bacterial growth which then compete with the algae for nutrients (Van den Meersche et al., 2004). Therefore, the survival of Bacteroidetes populations seems to be linked to environmental conditions and the physiological state of neighboring algae.

#### ABC Transporters Reveal Ecological Niches

In Alpha- and Beta-proteobacteria ATP-binding cassette (ABC) transporters were the most prevalent transport proteins identified (**Figure 4**). This is in agreement with previous freshwater and marine metaproteomic studies (Ng et al., 2010; Teeling et al., 2012; Georges et al., 2014). The majority of the ABC transporters were periplasmic-binding proteins (PBPs). The high representations of PBPs is commonly observed in aquatic metaproteomic studies. These subunits are far more abundant than the ATPase or permease components of ABC transporters in order to increase the frequency of substrate capture. Membrane proteins (e.g., permeases) are also inherently difficult to extract and solubilize therefore reducing the frequency of their detection (Williams and Cavicchioli, 2014).

In a metaproteomic comparison of Atlantic Ocean winter and spring microbial plankton, Georges et al. (2014) found ABC transporters were more abundant in low nutrient surface waters in mid-bloom and were mostly specific for organic substrates. Therefore, these type of transporters may be expected to more prevalent in the early oligotrophic conditions of our study where bacterial levels were higher (**Figure 1**) and the environment was rich in algal and cyanobacterial exudate (discussed in previous section). As expected, transporter proteins in Alpha- and Beta-proteobacteria were more abundant in oligotrophic than eutrophic conditions (emPAI = 3.32 and emPAI = 1.73, respectively). They were predominant in early phase in oligotrophic (early, emPAI = 1.42 and late, emPAI = 0.9) and late phase in eutrophic conditions (early, emPAI = 0.42 and late, emPAI = 0.88). Furthermore, in both treatments and timepoints the majority of ABC transporters were specific for organic substances (i.e., carbohydrates and amino acids). This suggests that both proteobacterial phyla are specialized in obtaining nutrients from DOM therefore investing more resources in the acquisition of organic rather than inorganic substrates and were favored in early oligotrophic when the rate of algal exudation was potentially higher (Teeling et al., 2012).

Finally, another particularity of ABC transporters is that the expression of these transporters comes at an additional metabolic cost and therefore they are mainly synthesized to target substrates that are limiting in the environment. Thus, determining which transporters are being expressed can provide clues to which substrate is limiting. There was a clear difference in substrate preference between the two (**Figure 4**); *Rhodobacter* sp. (Alpha-proteobacteria) carbohydrate transporter expression was more than twofold higher than amino acid transporter expression (carbohydrate, emPAI = 0.57; amino acid, emPAI = 0.21) whereas in the bacterial group *Hydrogenophaga* sp. (Beta-proteobacteria) only amino acid transporter expression was observed (carbohydrate, emPAI = 0.00; amino acid, emPAI = 0.81). This has been previously observed (Schweitzer et al., 2001; Pérez et al., 2015) and is a case of resource partitioning, a mechanism through which two phylogenetic groups can co-exist in the same environment without leading to competitive exclusion (Morin, 2011).

## Conclusion

A label-free comparative metaproteomics approach was applied on an experimental microcosm community under differing trophic states. The identification of proteins in early and late oligo- and eutrophic conditions allowed us to link function to phylogenetic diversity and reveal individual transitional niches. The results from this study also compared favorably with many *in situ* aquatic metaproteomic studies.

Algae and cyanobacteria predominantly expressed, as would be expected, proteins related to photosynthesis and carbon fixation. Interestingly, proteins involved in mechanisms of carbon concentration were abundant in virtually all samples, which indicated that carbon could be a limiting factor throughout the experiment. The fact that cyanobacteria, in eutrophic conditions, expressed several proteins related to environmental adaptation (e.g., microcompartment proteins) suggests that they may be better equipped than algal species to dominate nutrient enriched environments.

Proteins identified in all bacterial species suggested an alignment with oligotrophic environments. In early oligotrophic, Bacteroidetes showed characteristics that suggest a role as a fast-growing population that is specialized in cell and particle attachment and are the first to respond to algal growth. This ecosystem role can coexist with bacterial heterotrophs that live suspended in the water column and depend on algal exudate and decaying organic matter. ABC transporters were amongst the most abundant proteins identified. In a case of resource partitioning it was found that Alpha- and Beta-proteobacteria co-exist and metabolize algal/cyanobacterial exudate, but the former will preferentially uptake carbohydrates whereas the latter will prefer amino acid uptake thus avoiding direct competition. There is the evidence that bacterial metabolism controls primary production through the remineralization of nutrients, however, here it is shown that primary producers can also be a driver of bacterial community composition and function.

This study successfully showed that microcosms can be used to observe microbial mechanisms that are typical of the natural environment. While these microcosm systems are simplified, and may not completely represent global biogeochemical cycles, they can accurately provide a snapshot of a microbial community in controlled conditions, and offer the potential to employ more manipulative experimentation to uncover functions and processes in oligo- and eutrophic conditions. The study also demonstrated that a community metagenetic analysis can provide a usable database for high mass accuracy metaproteomics studies. Ultimately, these data suggest that nutrient enrichment affected the dynamics of individual microbes and how they interact with others in their vicinity. Further manipulative experiments and associated ‘omics’ methodology will significantly contribute to our understanding of how microbial communities adapt to local environmental conditions.

## Author Contributions

This study was designed and coordinated by DR, AB, and JP. JP, as principal investigator, and AB provided technical and conceptual guidance for all aspects of the project. DR planned and undertook all the practical aspects of the manuscript. NC contributed to performing and analyzing all aspects of protein extraction and analysis. The manuscript was written by DR and commented on by all authors.

## Conflict of Interest Statement

The authors declare that the research was conducted in the absence of any commercial or financial relationships that could be construed as a potential conflict of interest.

## Footnotes

1 http://proteomecentral.proteomexchange.org

2 http://www.uniprot.org/uploadlists/.

3 http://pfam.xfam.org/

4 http://eggnogdb.embl.de/.
