# Supplementary material for: A Metaproteomic Analysis of the Response of a Freshwater Microbial Community under Nutrient Enrichment
Source: Front Microbiol. 2016 Aug 3;7:1172. doi: 10.3389/fmicb.2016.01172 (PMC4971099; doi:10.3389/fmicb.2016.01172)
Supplement: Supplementary file 1 [file Table_1.DOCX]

Supplementary Table 1. Complete composition of artificial freshwater growth medium.

| Component | Concentration (mg L^-1^) |
| --- | --- |
| NaHCO_3_  MnCl_2._4H_2_O  MgSO_4_7H_2_O  KCl  H_2_SeO_3_  Ca(NO_3_)_2_4H_2_O  NH_4_Cl  KH_2_PO_4_  K_2_PO_4_  ZnSO_4_.7H_2_O  Na_2_EDTA.2H_2_O  H_3_BO_3_  FeSO4.7H_2_O  CuSO_4_.5H_2_O  CoCl_2_.6H_2_O  (NH_4_)_6_Mo7O_24_.4H_2_O | 192  0.18  115  0.45  0.0016  0.8  1  0.025  0.025  0.022  0.5  0.114  0.05  0.016  0.016  0.011 |
