# Supplementary material for: A Metaproteomic Analysis of the Response of a Freshwater Microbial Community under Nutrient Enrichment
Source: Front Microbiol. 2016 Aug 3;7:1172. doi: 10.3389/fmicb.2016.01172 (PMC4971099; doi:10.3389/fmicb.2016.01172)
Supplement: Supplementary file 2 [file Table_2.DOCX]

Supplementary Table 2. Complete list of the eukaryotic organisms in the experimental freshwater microbial community inoculum as determined by 18S rDNA sequencing.

| Eukaryotic organisms | |
| --- | --- |
| *Chloromonas pseudoplatyrhyncha* | %  26.93 |
| *Stephanodiscus sp* | 18.17 |
| Unsequenced organisms | 17.87 |
| *Chromulinaceae sp* | 8.48 |
| *Synedra angustissima* | 4.99 |
| *Ochromonadales sp* | 3.01 |
| *Chlamydomonas sp* | 2.98 |
| *Micractinium pusillum* | 1.62 |
| *Chlorella sp* | 1.08 |
| *Chloromonas cf schussnigii* | 0.58 |
| *Asterarcys quadricellulare* | 0.41 |
| *Cymbella minuta* | 0.38 |
| *Chloromonas insignis* | 0.30 |
| *Chlamydomonas reinhardtii* | 0.29 |
| *Chlamydomonas monadina* | 0.27 |
| *Chlorella fusca* | 0.26 |
| *Cyclotella meneghiniana* | 0.24 |
| *Paulschulzia pseudovolvox* | 0.16 |
| *Scenedesmus deserticola* | 0.11 |
| *Treubaria schmidlei* | 0.09 |
| *Choricystis sp* | 0.01 |
| *Chloroidium saccharophila* | 0.01 |
| *Oocystis marssonii* | 0.01 |
