# Supplementary material for: A Metaproteomic Analysis of the Response of a Freshwater Microbial Community under Nutrient Enrichment
Source: Front Microbiol. 2016 Aug 3;7:1172. doi: 10.3389/fmicb.2016.01172 (PMC4971099; doi:10.3389/fmicb.2016.01172)
Supplement: Supplementary file 3 [file Table_3.DOCX]

Supplementary Table 3. Complete list of the prokaryotic organisms in the experimental freshwater microbial community inoculum as determined by 16S rDNA sequencing.

| Prokaryotic organisms | |
| --- | --- |
| *Rhodoferax sp* | %  21.94 |
| Unsequenced organisms | 17.84 |
| *Flavobacterium sp* | 9.43 |
| *Anabaena sp* | 8.85 |
| *Brevundimonas diminuta* | 4.20 |
| *Hydrogenophaga sp* | 3.41 |
| *Runella limosa* | 2.47 |
| *Haliscomenobacter sp* | 2.43 |
| *Rhodobacter sp* | 2.34 |
| *Planktophila limnetica* | 2.13 |
| *Agrobacterium tumefaciens* | 2.11 |
| *Sphingobacterium sp* | 2.03 |
| *Ochrobactrum tritici* | 1.98 |
| *Brevundimonas variabilis* | 1.83 |
| *Sphingomonas sp* | 1.73 |
| *Curvibacter sp* | 1.48 |
| *Phenylobacterium falsum* | 1.42 |
| *Roseomonas stagni* | 1.24 |
| *Oceanicaulis sp* | 1.03 |
| *Erythromicrobium sp* | 0.89 |
| *Anabaena bergii* | 0.73 |
| *Rhodoluna lacicola* | 0.70 |
| *Inhella inkyongensis* | 0.60 |
| *Caulobacter sp* | 0.53 |
| *Sphingopyxis sp* | 0.51 |
| *Sphingopyxis alaskensis* | 0.43 |
| *Erythromicrobium ramosum* | 0.36 |
| *Achromobacter sp* | 0.33 |
| *Alcaligenes sp* | 0.32 |
| *Roseococcus sp* | 0.31 |
| *Albidiferax ferrireducens* | 0.30 |
| *Phormidium sp* | 0.29 |
| *Hyphomicrobium sp* | 0.28 |
| *Delftia sp* | 0.28 |
| *Bosea thiooxidans* | 0.28 |
| *Rhizobium sp* | 0.28 |
| *Prosthecomicrobium pneumaticum* | 0.26 |
| *Stenotrophomonas sp* | 0.24 |
| *Paucimonas lemoignei* | 0.21 |
| *Verrucomicrobium sp* | 0.21 |
| *Reyranella massiliensis* | 0.18 |
| *Afipia sp* | 0.18 |
| *Limnohabitans sp* | 0.14 |
| *Pedobacter sp* | 0.13 |
| *Polynucleobacter necessarius* | 0.13 |
| *Sandarakinorhabdus sp* | 0.12 |
| *Polaromonas sp* | 0.11 |
| *Rhodoferax antarcticus* | 0.11 |
| *Hahella sp* | 0.11 |
| *Devosia ginsengisoli* | 0.08 |
| *Devosia sp* | 0.06 |
| *Gluconobacter oxydans* | 0.06 |
| *Methylophilus sp* | 0.05 |
| *Rheinheimera sp* | 0.05 |
| *Synechococcus sp* | 0.04 |
| *Acetobacter sp* | 0.04 |
| *Planctomyces sp* | 0.03 |
| *Aquabacterium sp* | 0.03 |
| *Defluvibacter sp* | 0.02 |
| *Acidovorax sp* | 0.02 |
| *Mycobacterium sp* | 0.01 |
| *Sediminibacterium salmoneum* | 0.01 |
| *Methylocella sp* | 0.01 |
| *Mesorhizobium sp* | 0.01 |
| *Pseudomonas sp* | 0.01 |
| *Sediminibacterium sp* | 0.01 |
| *Polynucleobacter sp* | 0.01 |
| *Bacteriovorax sp* | 0.01 |
| *Pseudomonas putida* | 0.01 |
