# Supplementary material for: A Metaproteomic Analysis of the Response of a Freshwater Microbial Community under Nutrient Enrichment
Source: Front Microbiol. 2016 Aug 3;7:1172. doi: 10.3389/fmicb.2016.01172 (PMC4971099; doi:10.3389/fmicb.2016.01172)
Supplement: Supplementary file 4 [file Image_1.PDF]

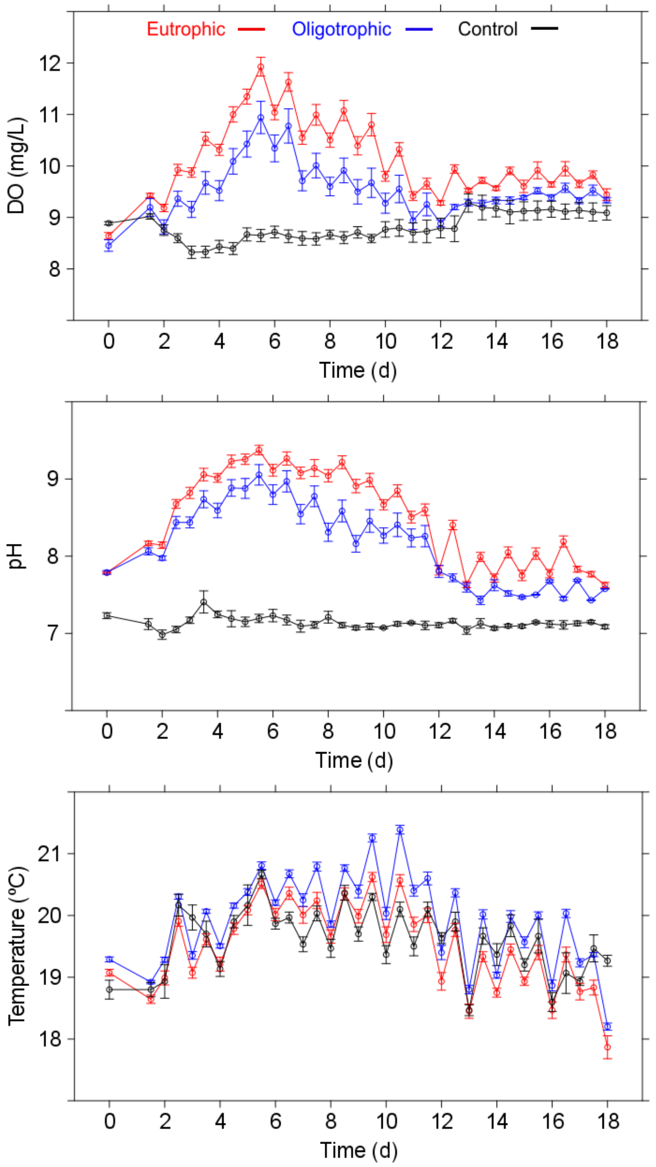

Supplementary Figure 1. Time series of the measured variables in the oligotrophic and eutrophic treatments. (A) Dissolved oxygen, (B) pH, (C) temperature, Legend is shown in the top panel. Error bars show standard errors (n = 3).
